# Tracing Pilots’ Situation Assessment by Neuroadaptive Cognitive Modeling

**DOI:** 10.3389/fnins.2020.00795

**Published:** 2020-08-11

**Authors:** Oliver W. Klaproth, Christoph Vernaleken, Laurens R. Krol, Marc Halbruegge, Thorsten O. Zander, Nele Russwinkel

**Affiliations:** ^1^Airbus Central R&T, Hamburg, Germany; ^2^Chair of Cognitive Modelling in Dynamic Systems, Department of Psychology and Ergonomics, Technische Universität Berlin, Berlin, Germany; ^3^Airbus Defence and Space, Manching, Germany; ^4^Zander Laboratories B.V., Amsterdam, Netherlands; ^5^Chair of Neuroadaptive Human-Computer Interaction, Brandenburg University of Technology, Cottbus-Senftenberg, Germany

**Keywords:** situation awareness, aviation, brain-computer-interfaces, ACT-R, human-automation interaction

## Abstract

This study presents the integration of a passive brain-computer interface (pBCI) and cognitive modeling as a method to trace pilots’ perception and processing of auditory alerts and messages during operations. Missing alerts on the flight deck can result in out-of-the-loop problems that can lead to accidents. By tracing pilots’ perception and responses to alerts, cognitive assistance can be provided based on individual needs to ensure they maintain adequate situation awareness. Data from 24 participating aircrew in a simulated flight study that included multiple alerts and air traffic control messages in single pilot setup are presented. A classifier was trained to identify pilots’ neurophysiological reactions to alerts and messages from participants’ electroencephalogram (EEG). A neuroadaptive ACT-R model using EEG data was compared to a conventional normative model regarding accuracy in representing individual pilots. Results show that passive BCI can distinguish between alerts that are processed by the pilot as task-relevant or irrelevant in the cockpit based on the recorded EEG. The neuroadaptive model’s integration of this data resulted in significantly higher performance of 87% overall accuracy in representing individual pilots’ responses to alerts and messages compared to 72% accuracy of a normative model that did not consider EEG data. We conclude that neuroadaptive technology allows for implicit measurement and tracing of pilots’ perception and processing of alerts on the flight deck. Careful handling of uncertainties inherent to passive BCI and cognitive modeling shows how the representation of pilot cognitive states can be improved iteratively for providing assistance.

## Introduction

Irrespective of ubiquitous automation, current-generation commercial and business aircraft still rely on pilots to resolve critical situations caused, among others, by system malfunctions. Pilots need to maintain situational awareness (SA) so they can assume manual control or intervene when necessary. It is essential for flight safety that pilots understand the criticality of flight deck alerts, and do not accidentally miss alerts, e.g., due to high workload and cognitive tunneling (Dehais et al., 2014). Human-machine interfaces on the flight deck therefore need to ensure messages are processed correctly to reduce the risk of out-of-the-loop problems (Endsley and Kiris, 1995; Berberian et al., 2017). Failed, delayed or otherwise inadequate response to flight deck alerts has been associated with several fatal accidents in the past (Air Accident Investigation and Aviation Safety Board, 2006; Aviation Safety Council, 2016).

Automation has transformed pilots’ role from hands-on flying to monitoring system displays which is ill-matched to human cognitive capabilities (Bainbridge, 1983) and facilitates more superficial processing of information (Endsley, 2017). Furthermore, reduced-crew (e.g., single-pilot) operations can increase demands on pilots in commercial aircraft through elevated workload of remaining crew (Harris et al., 2015) and higher complexity imposed by additional automation (Bailey et al., 2017). More complex automation can impede the detection of divergence in the situation assessment by human operator and automated system, neither of which may adequately reflect reality (Rußwinkel et al., 2020). We believe that neurotechnologies can be used for cognitive enhancement and support of pilots in face of increased demands (Scerbo, 2006; Cinel et al., 2019). One way to achieve this is by monitoring the pilots’ cognitive states and performance during flight deck operations in order to detect the onset of such divergence e.g., cognitive phenomena that may lead to out-of-the-loop situations. Being able to detect such cognitive states, corrective measures may be initiated to prevent or reduce risk of out-of-the-loop situations and to maintain the high level of safety in aviation.

### OOTL and Situation Awareness

Out-of-the-loop problems arise when pilots lack SA (Endsley and Jones, 2011). SA is progressively developed through the levels of perception (1), comprehension (2), and projection (3) of a situation’s elements. Missing critical alerts impairs situation perception and inhibits the development of higher SA levels. In a study on pilot errors, the vast majority of errors could be accounted to incorrect perception (70.3%) and comprehension (20.3%) of situations (Jones and Endsley, 1996).

Situational awareness is commonly measured by sampling with the help of probing questions. Probes can give insights into pilots’ deeper understanding of a situation as well as whether or not a probed piece of information can be retrieved from memory. However, probing methods either require flight scenarios to be frozen (e.g., Endsley, 2000) or incur extra workload (Pierce, 2012) when assessing pilots’ SA. Physiological (e.g., Berka et al., 2006; van Dijk et al., 2011; Di Flumeri et al., 2019) and performance-based metrics (e.g., Vidulich and McMillan, 2000) are less direct measures of memory contents, but they can be used unobtrusively in operations (see Endsley and Jones, 2011, for a summary of measures). As an example, van Dijk et al. (2011) showed how eye tracking can serve as an indicator of pilots’ perceptual and attentional processes. The abundance of visual information in the cockpit, however, makes tracing visual attention very challenging and susceptible to selective ignoring and inattentional blindness (Haines, 1991; Most et al., 2005).

Alerts in the cockpit are presented both visually and acoustically, while acoustic stimuli have shown to be more effective in attracting attention (Spence and Driver, 1997). Physiological responses to alert stimuli may reveal whether or not alerts have been perceived and processed. For example, event-related potentials (ERPs) in operators’ electroencephalogram (EEG) were proposed as indicators of attended and unattended stimuli in the assessment of SA (Endsley, 1995). Dehais et al. (2016) demonstrated that ERP components indeed allow to differentiate between missed and processed auditory stimuli in the cockpit, even in single trials (Dehais et al., 2019). They noted that these differences are primarily reflected in early perceptual and late attentional stages of auditory processing. According to Dehais et al. (2019), failure to adequately perceive or process an alert is likely due to excessive demand to cognitive resources in terms of attention and memory at a central executive level. In addition, deterministic modeling individual processed or missed alerts requires lots of data about the situation and the pilot’s state and neurophysiological measures can help reduce uncertainty.

Thus, by monitoring what stimuli are provided when and checking for ERPs at stimulus onset, perception of a situation could be tracked in real-time (Wilson, 2000). After that, performance metrics in terms of comparing pilots’ actual behavior to normative procedures can provide information on later SA stages. In contrast to product-focused measures, this process-based approach of situation assessment (Sarter and Sarter, 2003; Durso and Sethumadhavan, 2008) allows to also capture implicit components of SA (Endsley, 2000) that might be overlooked in SA probing.

### Requirements for Cognitive State Assessment

As cognitive states underlying situation assessment are not directly observable, their detection and prediction in this study is approached from different angles by neurophysiological measures and cognitive modeling. Consistent monitoring of a pilot’s situation assessment in flight requires tracing what elements of a situation are perceived and processed. Tracing perceptual and cognitive processing can best be done implicitly by interpreting psycho-physiological measures so as not to increase the pilots’ load or otherwise interfere with operations. As we are interested in event-related cognitive processing, i.e., the processing of specific visual or auditory alerts, one requirement is that the onset of these alerts is captured accurately (Luck, 2014). This allows the timing of each alert to be synchronized with a measurement of the pilots’ neuroelectric activity, which is sensitive to even slight temporal misalignments. This activity can then be analyzed relative to each alert’s exact onset, allowing alert-specific cognitive states to be decoded. Such automated, non-intrusive detection of cognitive processing can be done using a passive brain-computer interface (pBCI), based on a continuous measurement of brain activity (Zander and Kothe, 2011; Krol et al., 2018).

If unprocessed alerts are detected, cognitive assistance can be offered depending on the alert’s significance for the course of the operation. In order to assess the significance of a missed alert, its impact on SA and the operation can be simulated. This way, critical drops in pilot performance can be anticipated and assistance can be provided to prevent the pilot from getting out of the loop. This simulation can be performed using cognitive models that capture the characteristics of the human cognitive system such as resource limitations.

### Cognitive Pilot Models

ACT-R^1^ (Anderson et al., 2004) is the most comprehensive and widely used architecture to build models that can simulate, predict, and keep track of cognitive dynamics. It is based on accumulated research about the human brain’s modular architecture, where each module maps onto a different functional area of the brain. In its current 7.14 version the ACT-R architecture comprises separate modules for declarative and procedural memory, temporal, and intentional (i.e., “goal”) processing and visual, aural, motor, speech modules for limited perceptual-motor capabilities. While highly interconnected within themselves, exchange of symbolic information between modules is constrained by a small number of interfaces that are modeled as buffers (Anderson, 2007)^2^. These intermodular connections meet in the procedural memory module (representing the caudate of the basal ganglia; Anderson et al., 2008), where condition-action statements (i.e., “productions”) are triggered depending on buffer contents. Actions can be defined for example in terms of memory retrieval, directing attention or manipulating the outside world through speech or motor actions. Based on sub-symbolic mechanisms such as utility learning, spreading activation, memory decay, and random noise, ACT-R models can adapt to dynamic environments and represent average human behavior in non-deterministic fashion.

ACT-R has frequently been used for modeling pilots’ cognitive dynamics (e.g., Byrne and Kirlik, 2005; Gluck, 2010; Somers and West, 2013). It allows for the creation of cognitive models according to specific task descriptions, e.g., a goal-directed hierarchical task analysis (HTA; Endsley, 1995; Stanton, 2006). When this task description focuses on maintaining good SA, a normative cognitive model can be developed that acts in order to optimize SA. Normative models can be compared to individual pilot behavior to detect deviations and to make inferences about individual pilots’ SA. Tracing individual behavior (model-tracing; Fu et al., 2006) can suffer from epistemic uncertainty (Kiureghian and Ditlevsen, 2009), for example, when it is unknown why a pilot did not react to an alert. This uncertainty can be reduced by using physiological data alongside system inputs to build richer models of individual performance (Olofsen et al., 2010; Putze et al., 2015; Reifman et al., 2018). However, sensor data inaccuracies can introduce a different, aleatory kind of uncertainty that is hard to assign to individual observations and needs to be considered in design of adaptive models (Kiureghian and Ditlevsen, 2009).

ACT-R has gained popularity in modeling human autonomy interaction. The work of Putze et al. (2015) showed how an ACT-R model allows to modulate interface complexity according to operator workload measured in EEG. Ball et al. (2010) have developed a synthetic teammate able to pilot unmanned aerial vehicles and communicate with human teammates based on an extensive model of SA (see also Rodgers et al., 2013; Freiman et al., 2018). Both these models demonstrate how selected human capabilities such as piloting and communicating (McNeese et al., 2018) or being empathic to operators’ cognitive state (Putze et al., 2015) can be allocated to an ACT-R model in human autonomy teaming.

### Neuroadaptive Technology

Neuroadaptive technology refers to technology that uses cognitive state assessments as implicit input in order to enable intelligent forms of adaptation (Zander et al., 2016; Krol and Zander, 2017). One way to achieve this, is to maintain a model that is continuously updated using measures of situational parameters as well as the corresponding cognitive states of the user (e.g., Krol et al., 2020). Adaptive actions can then be initiated based on the information provided by the model. Cognitive states can be assessed in different ways. Generally, certain cognitive states result, on average, in specific patterns of brain activity, and can be inferred from brain activity if the corresponding pattern distributions are known. As patterns differ to some extent between individuals and even between sessions, it is usually necessary to record multiple samples of related brain activity in order to describe the pattern distribution of cognitive responses in an individual. Given a sufficient amount of samples of a sufficiently distinct pattern, a so-called classifier can be calibrated which is capable of detecting these patterns in real time, with typical single-trial accuracies between 65 and 95% (Lotte et al., 2007).

Importantly, since these cognitive states occur as a natural consequence of the ongoing interaction, no additional effort is required, nor task load induced, for them to be made detectable. It is thus possible to use a measure of a user’s cognitive state as implicit input, referring to input that was acquired without this being deliberately communicated by the operator (Schmidt, 2000; Zander et al., 2014). Among other things, this has already been used for adaptive automation. For example, without the pilots explicitly communicating anything, a measure of their brain activity revealed indices of e.g., engagement or workload, allowing the automation to be increased or decreased accordingly (e.g., Pope et al., 1995; Bailey et al., 2003; Aricò et al., 2016).

In the cockpit, each alert can be expected to elicit specific cortical activity, e.g., an ERP. If this activity can be decoded to reveal whether or not the alert has been perceived, and potentially whether and how it was processed, it can be used as implicit input. Since such input can be obtained from an ongoing measurement of the pilots’ brain activity, no additional demands are placed on the pilots. By interpreting this information alongside historic pilot responses and further operational parameters, an informed decision can be made about the current cognitive state of the pilots and recommended adaptive steps.

### Current Study

The remainder of this article describes the implementation and application of a concept for tracing individual pilots’ perception and processing of aural alerts based on neuroadaptive cognitive modeling. In contrast to conventional measures of SA, this method is designed for application in operations that require unobtrusive tracing of cognitive states. The method is applied to explore how to anticipate pilot behavior and when to offer assistance according to their cognitive state. To this end, we test (1) the feasibility of distinguishing between processed and missed alerts based on pilots’ brain activity, (2) whether individual pilot behavior can be anticipated using cognitive models, and (3) how the methods of pBCI and cognitive modeling can be integrated. Results are discussed regarding their implications for cognitive assistance on the flight deck and potential benefits for single pilot operations. Limitations are addressed to explore what else is needed in cognitive assistance for the anticipation and prevention of out-of-the-loop situations.

## Materials and Methods

This research complied with the American Psychological Association Code of Ethics and was approved by the Institutional Review Board at TU Berlin. Informed consent was obtained from each participant.

### Participants

Twenty-four aircrew (one female) with a mean age of 49.08 years (SD = 6.08) participated in the flight simulator study. Participants were predominantly military pilots with an average experience of 3230 h of flight (SD = 2330.71), of which on average 51.21 h (SD = 90.76) were performed in the previous year. All participating aircrew had normal or corrected to normal vision, all but two were right-handed.

### Procedure

Participating aircrew were asked for information on their flight experience and physical health relevant for physiological data assessment in the simulator. After application of EEG sensors, participants performed a desktop-based auditory oddball training paradigm (Debener et al., 2005). Participants performed 10 blocks during each of which a sequence of 60 auditory tones was presented. Each tone could be either a standard tone of 350 Hz occurring 70–80% of the time, a target deviant tone of 650 Hz (10–15%), or non-target deviant (2000 Hz, 10–15%). There was a variable interval between stimulus onsets of 1.5 ± 0.2 s, and a self-paced break after each block. Each tone lasted 339 ms. Participants were instructed to count the target tones in each block with eyes open, and to verbally report their count after each block to ensure they stayed attentive during the task. Thus, the standard tones represent frequent but task-irrelevant events, target tones represent rare task-relevant events, and the deviants were rare but task-irrelevant.

Following this, participants were seated in the simulator and briefed on the flying task. For the flight scenario, participants were instructed to avoid communicating with the experimenter during the scenario but were allowed to think aloud and to perform readbacks of air traffic control (ATC) messages just as they would during a normal flight. After the scenario, a debriefing session was conducted in order to collect feedback from participants.

### Simulator and Scenario

Participants flew a mission in the fixed-base cockpit simulator of a mission aircraft similar to current-generation business jets certified according to EASA CS-23, which may be operated by a single pilot. The mission was implemented and simulated using the open source flight simulation software “FlightGear 3.4”^3^. Participants’ task was to perform a fictitious routine VIP passenger transport from Ingolstadt-Manching (ETSI) to Kassel (EDVK) airport. To keep workload levels associated with basic flying low, the scenario started with the aircraft already airborne at cruise flight level (FL 250) with autopilot (altitude and NAV^4^ mode) engaged. According to the flight management system (FMS) flight plan presented, the remaining flight time was approximately 40 min in fair weather conditions. To maintain speed, thrust had to be adjusted manually, since the aircraft was – like most business jets today – not equipped with auto-thrust. To simulate interactions with ATC and to ensure a consistent flow of the scenario for all participants, pilots were presented with pre-recorded routine ATC instructions relating to flight level and heading changes at fixed time intervals after the start of the scenario.

Also, at pre-defined times, pilots would encounter a series of flight deck alerts of varying, but generally increasing severity. First, 4 min into the scenario, the main fuel pump in the right wing tank failed, resulting in a caution level flight deck alert and, subsequently, the display of a simple recovery procedure, which was automatically presented as electronic checklist. After 6 min, a small fuel leak appeared in the right fuel tank, which had initially no salient flight deck effects and would therefore go mostly unnoticed. Contributing to this was a TCAS traffic advisory (caution level alert) after approximately 7 min, which would coincide with an ATC instruction to descend due to traffic (e.g., “F-UO^5^, due to traffic, descend and maintain FL 280” or “F-UO, direct TUSOS and descend FL 200”). Moreover, to simulate the effects of an intermittent spurious alert, and to divert pilot attention from the FUEL format to decrease the chance of the pilot noticing the leak, an identical caution-level alert of an electrical bus system failure was triggered four times throughout the scenario. This alert would automatically be removed after 5 s without any pilot action, and before pilots were able to access the associated recovery procedure. When the fuel leak had caused a fuel imbalance exceeding a certain threshold, a caution-level alert relating to the imbalance would be raised. The associated procedure would then guide pilots through several steps intended to find the root cause of the fuel imbalance. The scenario ended once an in-flight fire of the left engine initiated after 16:40 min, resulting in a warning level alert, had successfully been extinguished by the pilot. To make sure that all participants encountered all events of the scenario, speed warnings were issued dynamically by the simulated ATC whenever airspeed did not remain within a predefined range.

**FIGURE 1:**
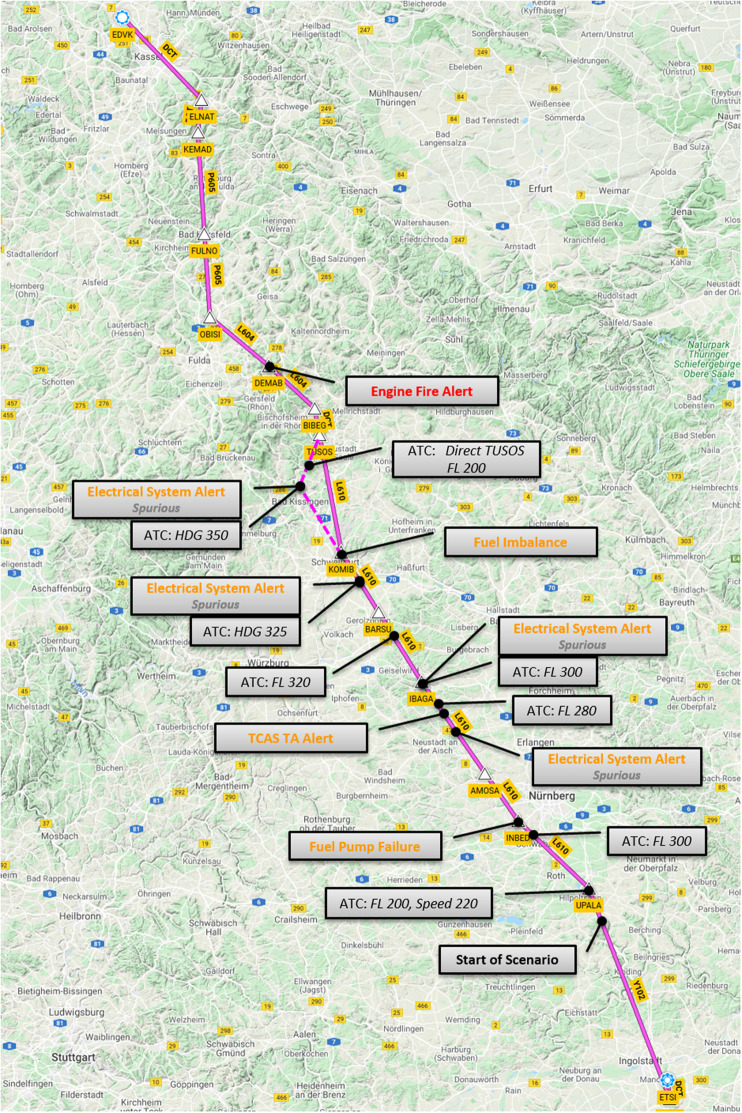
Lateral profile of simulator task including events, waypoints, and geographic information.

**FIGURE 2:**
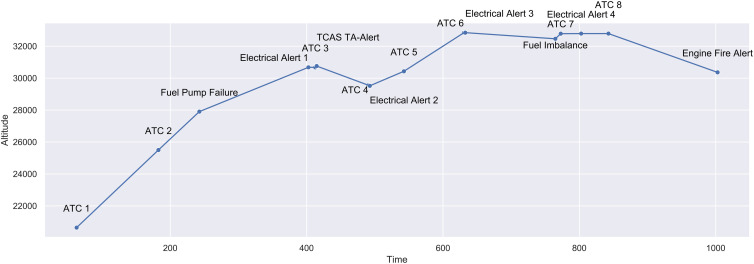
Vertical profile of simulator task including events, altitude in meters, and timing in seconds.

Figure 1 gives an overview of events’ position on the flight path while Figure 2 shows the vertical profile including timing of events during the flight task. Normative responses to these events would result in the following respective parameter changes:

• ATC 1: Altitude-Select 280 and Speed-Select 220.
• ATC 2: Altitude-Select 300.
• Fuel Pump Failure: Right-Main-Pump Off.
• Electrical Systems Alert 1: No parameter change^6^.
• ATC 3: Altitude-Select 280.
• TCAS TA-Alert: No parameter change.
• ATC 4: Altitude-Select 300.
• Electrical Systems Alert 2: No parameter change.
• ATC 5: Altitude-Select 320.
• ATC 6: Heading-Select 325.
• Electrical Systems Alert 3: No parameter change.
• Fuel Imbalance: Fuel-X-Feed True (not included in data analysis).
• ATC 7: Heading-Select 350.
• Electrical Systems Alert 4: No parameter change.

### EEG

Electroencephalogram was recorded continuously at 500 Hz using a mobile, wireless LiveAmp amplifier (Brain Products, Gilching, Germany) using 32 active Ag/AgCl electrodes arranged on actiCAP caps according to the international 10–20 system and referenced to FCz. EEG was synchronized with both the desktop stimuli and the flight events using the Lab Streaming Layer (Kothe, 2014) software framework to ensure that EEG data could be related to the respective simulator events with adequate temporal resolution. In particular, FlightGear was configured to log the status of each of the alarms and send it at 100 Hz to a UDP port, where a custom Python script listened for incoming data and immediately forwarded each packet through LSL. A change in alert status could then be interpreted as the on- or offset of the alert.

### ERP Classification

A windowed-means classifier (Blankertz et al., 2011) was calibrated on the EEG data recorded for each individual participant during the oddball paradigm to distinguish between their neurophysiological response to two different categories of tones. Features were the mean amplitudes of eight consecutive non-overlapping time windows of 50 ms each starting at 150 ms following onset of the auditory tone, after band-pass filtering the signal between 0.3 and 20 Hz. Shrinkage-regularized linear discriminant analysis was used to separate the classes. A fivefold cross-validation with margins of five was used to obtain estimates of the classifier’s parameters and accuracy. We focused on distinguishing between standard versus target tones, i.e., task-irrelevant versus task-relevant events. The classification algorithm was implemented using BCILAB (Kothe and Makeig, 2013).

The trained classifier was optimally capable of distinguishing between the two categories of tones based solely on the participant’s brain activity following each tone’s onset. Having trained the classifier on detecting differences between these events in an abstract oddball task, we then applied the classifier to the data recorded during that same participant’s flight. This thus allowed us to investigate to what extent flight deck alerts could be reliably identified as the comparable equivalent of “standard” (task-irrelevant, unimportant) or “target” (etc.) tones, based solely on the pilots’ EEG data less than 1 second after onset of each event. For each simulated flight event, the classifier returned a number between 1 and 2, signifying that the neurophysiological response was closest to the activity following standard (1) or target (2) tones in the oddball paradigm, respectively.

### Cognitive Model

A normative and a neuroadaptive cognitive model were created following a HTA performed with a subject matter expert for the flight scenario using ACT-R. For the HTA and the cognitive model, good SA level 1 was defined as perceiving and paying attention to all auditory stimuli provided in the scenario. While adequacy of responses depended on the type of alert or contents of ATC messages, the time limit for initiating a first reaction to an alert was set to 25 s for all events. As the spurious electrical bus alerts disappeared before pilots were able to react, they are not included in the analysis of this article. The interface between the models and the simulator/Flight Gear was implemented as an extended version of ACT-CV (Halbrügge, 2013), where log files of cockpit system states recorded with a sampling rate of 20 Hz served as ACT-R task environment.

Both normative and neuroadaptive model were based on a routine loop consisting of monitoring flight parameters and managing thrust accordingly in order to have comparable workload as participants in the simulator; however, cognitively plausible modeling of workload and accuracy in thrust management was beyond the focus of this study and therefore not evaluated. The routine loop was temporarily exited when an aural alert was perceived. The normative model shifted its attention to read the warning message and initiate the corresponding procedure.

In order to illustrate the model’s flow of information from one module to another with respect to ACT-R’s neuroanatomical assumptions, associated brain areas as described by Anderson et al. (2008) and Borst et al. (2013) will be given in parentheses behind each module. The validation of activity predicted by the model with brain imaging data was beyond the scope of this article. For example of the fuel pump failure alert the model would go through the following steps: (1) a chunk representing a sound activates the aural module (mapped to the superior temporal gyrus) by being put in the model’s aural-location buffer. (2) Next, this information allows the procedural module (basal ganglia) to fire a production that starts counting seconds passed since the alert with the temporal module and that decodes the sound as an alert sound using the aural buffer. This latter information would trigger productions that (3) make the model shift its visual attention to the warning display by calling on the visual module’s (fusiform gyrus) visual-location buffer and (4) read the written fuel pump failure message using the visual buffer. (5) The following production would result in calling up the corresponding pump failure checklist, memorizing its first item (i.e., pressing the right main fuel pump pushbutton) in the imaginal buffer (intraparietal sulcus, representing the model’s short-term memory problem state). (6) Then, using its motor module (precentral gyrus), the model acts as if pressing the pump pushbutton (without changing any of the flight parameters) before (7) reading and carrying out the remaining checklist items in the same fashion while it keeps counting. (8) Finally when the count in the temporal module has reached 25 s, the module checks the flight parameters for the state of the right main fuel pump’s pushbutton to verify whether the pilot has carried out the action required by the first checklist item as memorized in the model’s imaginal buffer.

As the normative model assumed that pilots will correctly process each alert, adequate responses were scored as correct and inadequate (i.e., commission errors) as well as lacking and too late responses (i.e., omission errors) as incorrect classification of behavior. Adequacy and timeliness of responses were scored according to criteria assessed in the HTA with subject matter experts. For example, if an ATC message requested a flight level change to 300, entering an altitude-select of 300 in the flight control unit within a time window of 25 s was scored as good performance; all other responses such as entering an altitude-select of 280 or entering the correct altitude-select after 25 s were classified as missed ATC message. The fraction of incorrect classifications was treated as epistemic uncertainty (μ_Epistemic_) as the model had no information about why the pilot did not respond as expected.

The neuroadaptive model considered individual brain activity when classifying behavior to reduce this uncertainty. pBCI data were provided to the model along with the cockpit systems data. After each acoustic alert and message was decoded, the neuroadaptive model checked if the sound was processed as task-relevant by the participant according to pBCI data before shifting its visual attention to read the alert’s or message’s actual content. To build and improve on the normative model’s accuracy, the neuroadaptive model assumed that alerts will be processed correctly. If pBCI data showed that a message was processed as irrelevant (classifier output <1.5), the model scored lacking or inadequate responses as correct behavior classification. If the message was processed as relevant but no adequate response can be found, the model scored its classification as incorrect and treats these cases as epistemic uncertainty.

Responses were assessed for 10 events for each of the 21 pilots whereof eight ATC messages, one amber, and one red alert. Model accuracies were computed across participants as the fraction of correct classifications in all events. Normative and neuroadaptive model were compared by a paired samples *t*-test. Effect size is reported as Cohen’s *d*_av_ (Lakens, 2013). Aleatory uncertainty (μ_Aleatory_) was defined as one minus EEG classifier accuracy. Though aleatory uncertainty affects correct and incorrect classifications, an accuracy corrected for aleatory uncertainty was computed for the neuroadaptive model. The distribution of lacking and inadequate responses was tested for a relationship with EEG classifications by a Chi-square test. A detailed description of the cognitive model including the overall approach and modeling decisions made can be found in Klaproth et al. (2020).

## Results

### ERP Classification

**FIGURE 3:**
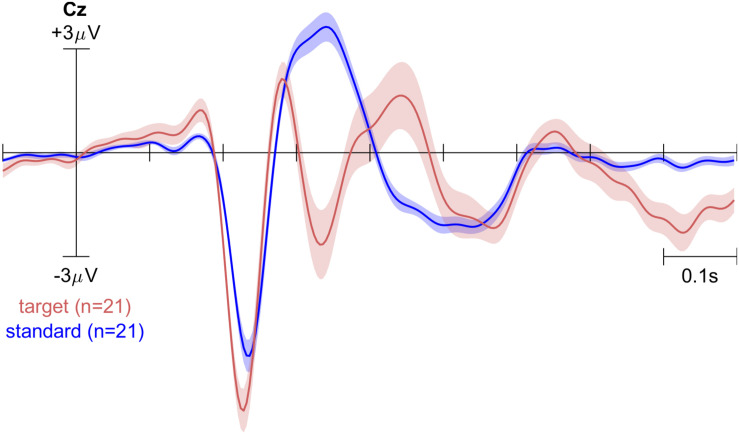
The grand-average ERP of 21 participants showing responses to target and standard tones on channel Cz. Shaded area indicates the standard error of the mean.

Figure 3 shows the grand-average ERPs on channel Pz for the standard and target tones during the oddball experiment on three electrode sites. Note that there is a delay. We had previously estimated our stimulus presentation pipeline to contain a lag of approximately 150 ms. This would coincide with the common interpretation that the initial negative peak visible in these plots is the N100.

The classifier was trained to detect the differences between single-trial ERPs using all 32 channels and had a cross-validated averaged accuracy of 86%. Given the class imbalance between the standard deviant tones, chance level was not at 50% for this binary classifier. Instead, significant classification accuracy (*p* < 0.05) is reached at 78%. The classes could be separated with significant accuracy for all but three participants. This was in part due to technical issues with the EEG recording. These three participants were excluded from further analysis.

The classifier trained on data from the oddball paradigm was subsequently applied to data following four flight events: ATC messages, the spurious electrical bus system failure alert, the fuel imbalance alert, and the fire alert. These classification results provided information to be used in the neuroadaptive cognitive model.

### Cognitive Model

**FIGURE 4:**
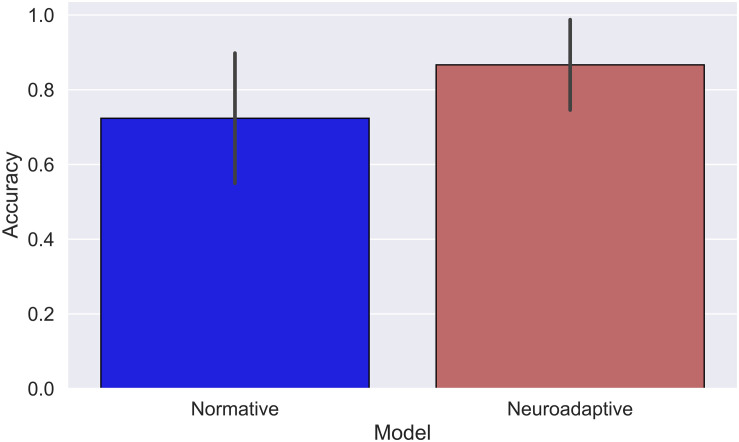
Mean model accuracies, error bars indicate standard deviations across participants.

**FIGURE 5:**
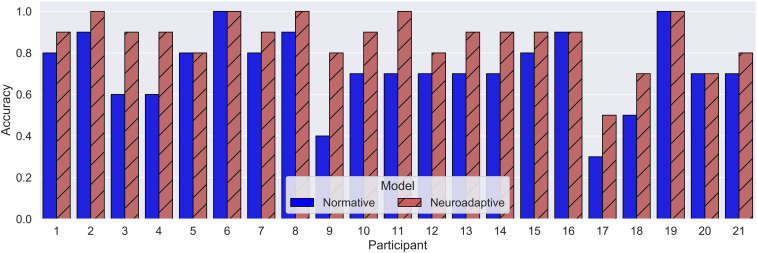
Mean model accuracies per model and participant.

The normative model correctly described participants’ behavior for 162 of the total 210 observed events (*M*_Normative_ = 0.72, SD = 0.09), indicating that participants missed to respond to 48 events. The neuroadaptive model was able to simulate 182 of participant’s responses correctly (*M*_Neuroadaptive_ = 0.87, SD = 0.13, see Figure 4), resulting in a significant added value of including pBCI data compared to the normative model [*t*(20) = 5.62, *p* < 0.01, *d*_av_ = 1.3]. Figure 5 shows the respective models’ accuracies for each of the 21 pilots.

Epistemic uncertainties for the models are μ_Epistemic_ = 0.28 for the normative and μ_Epistemic_ = 0.13 for the neuroadaptive model. The added value of the neuroadaptive over the normative model is 0.15, so the neuroadaptive model’s accuracy corrected for EEG-classifier accuracy of 0.88 is 0.85 with μ_Epistemic_ = 0.15 and μ_Aleatory_ = 0.02.

Of the 58 events left unexplained by the normative model, 22 events did not show a response to the respective alert or message and 36 showed an incorrect response by the participant. Chi-square tests yielded no significant relationship between EEG classifier output (standard/target) and the event having missing or incorrect responses [χ^2^(1, *N* = 58) = 1.04, *p* = 0.31), i.e., pBCI-data do not predict whether a participant will respond incorrectly or not at all to missed alerts.

## Discussion

The use of increasingly complex and less traceable automation can result in out-of-the loop situations thanks to different assessment of situations by pilot and automated system. Results of this study have demonstrated the feasibility of implicitly detecting and handling of emerging divergence in situation assessment with the help of a neuroadaptive cognitive model.

Using a pBCI for real-time assessment of cognitive responses evoked by events in the cockpit provides insight into subjective situational interpretations. Such information is highly dependent on the context sensitive, individual state of the operator and can hardly, if at all, be inferred by purely behavioral or environmental measures. In general, we conclude that the combination of pBCI approaches with advanced methods of cognitive modeling, leads to an increase in the reliability and capability of the resulting cognitive model – introducing the idea of neuroadaptive cognitive modeling – as shown in this study.

Specifically, the ERP produced by the oddball paradigm shows clear differences between the different categories of tones. In particular, a P300 at Pz clearly distinguishes between target (task-relevant) and standard (task-irrelevant) tones. Based on these differences in single-trial event-related activity, the classifier was capable of distinguishing between target and standard tones with single-trial accuracies significantly higher than chance in the training session.

The improvement in the cognitive model that resulted from including the pBCI output indicates that it is possible to obtain informative cognitive state information based on a pilot’s brain activity immediately following an auditory event. The fact that the classifier decoding this information was trained in a desktop setting demonstrates that no elaborate training sessions are required.

Normative model results suggest that individual pilot behavior can be traced and anticipated by a cognitive model. By comparing individual pilots’ actions to the normative model behavior, deviations could be detected and inferences about SA could be made without intruding the task (Vidulich and McMillan, 2000). Twenty-eight percent of epistemic uncertainty, with lacking and incorrect responses evenly distributed, indicate that additional diagnostic information is required for meaningful analysis and support in cases of deviating behavior.

The improvement in accuracy for the neuroadaptive model demonstrate how individual behavior models can benefit from the integration of physiological data. Not only can top-down modeling of human cognition in a task be complemented by bottom-up integration of (neuro-) behavioral data for example to account for behavioral moderators (e.g., Ritter et al., 2004), it can also provide contextual information required for situation-dependent interpretation of EEG data. The different types of uncertainties inherent to model tracing and pBCI determined the model’s systematic design: pBCI data could only be used to reduce the fraction of the normative model’s unexplained behavior to deal with aleatory uncertainty.

The method’s limitations are quantified in terms of uncertainty. Later SA stages need to be monitored to increase accuracy in pilot modeling. Measures of additional physiological indicators might be connected in line to further reduce both epistemic uncertainty with new types of information, and aleatory with joint probability distributions. For example, gaze data such as visual search behavior in response to alerts could be indicative of comprehension problems and reinforce or challenge pBCI classifications of alerts being perceived or not. Other indicators, for example the error-related negativity component of the ERP, could help to identify situations where operators have low comprehension or are out of the loop (Berberian et al., 2017).

Any cockpit application of passive BCI technology requires a thorough consideration regarding the intrusiveness of the measurement, the intended function(s) enabled by the BCI, as well as the safety and airworthiness implications associated with this function. The intrusiveness perceived by pilots will mainly depend on how well the (dry) EEG electrodes can be integrated for example into the interior lining of a pilot helmet or the headband of a headset. The intended cockpit (assistance) function, in turn, will mainly determine the airworthiness certification and associated validation effort required.

If the system described in this article is merely be used to enhance the efficiency of the already certified flight deck alerting system of an aircraft, the design assurance level required from an airworthiness and safety perspective could be lower compared to a solution where a passive BCI-based cockpit function is an integral part of the aircraft’s safety net. In the latter case, the airworthiness effort will be substantial irrespective of whether AI and/or machine learning are used or not. Although evaluated offline after data collection, the methods presented in this paper are well-suited to be applied online without substantial modifications. While the abstract oddball task can replace more realistic alternatives to gather training data, and thus substantially shorten the amount of time required to do so, it may still be necessary to gather new training data before each flight due to the natural non-stationarity present in EEG activity. For a truly walk-up-and-use neuroadaptive solution, a subject-independent classifier would be required (e.g., Fazli et al., 2009). Monitoring pilots’ ERPs in response to alerts gives diagnostic value. Detection of inattentional deafness in early, perceptual ERP components could trigger communication of the alert in alternative modalities (e.g., tactile or visual; Liu et al., 2016). For unattended alerts detected in later ERP components, cockpit automation could prioritize and choose to postpone reminders in case of minor criticality. Withholding information that is not alert-related can be effective in forcing pilots’ attention onto the alert, but it may be accompanied by decrease in pilots’ authority and associated risks, for example to resilience in unexpected situations and technology acceptance.

The simulator setting likely introduced biases in task engagement and density of events in the scenario. Measuring system input from pilots while they monitor instruments in real flight conditions may not provide enough data to make inferences about cognitive states. This emphasizes the need for additional behavioral measures (e.g., neurophysiological activity, speech, or gaze) to provide individual assistance.

Pilots are capable of anticipating complex system behavior but reports of automation surprises and out-of-the-loop situations stress the importance of a shared understanding of situations by pilot and cockpit automation. Increasing complexity of automation should therefore go together with a paradigm shift toward human-autonomy teaming based on a shared understanding of the situation. This includes bi-directional communication whenever a significant divergence in the understanding of a situation occurs to provide information missing for shared awareness of the human autonomy team (Shively et al., 2017). Anticipation of divergences and understanding human information needs to ensure shared awareness remains a challenge for human autonomy teaming (McNeese et al., 2018). By addressing divergences in human and autonomy situation assessment, critical situations might be prevented or at least resolved before they result in incidents or accidents. Tracing pilots’ perception of cockpit events represents a first step toward this goal.

## Conclusion

A pBCI allows to implicitly monitor whether pilots have correctly processed alerts or messages without intruding the mission using a classifier trained in a desktop setting. The integration of pBCI data in cognitive pilot models significantly improves the accuracy in following up with pilots’ situation assessment. Tracing pilots’ situation assessment through neuroadaptive cognitive modeling may facilitate the early detection of divergences in situation assessment in human autonomy teams. While sensor obtrusiveness and computational limitations may obstruct application, neuroadaptive cognitive modeling could help to tracing of pilots’ situation awareness and enable adaptive alerting.

## Data Availability Statement

The datasets presented in this article were mainly collected using aircrew employed by Airbus. For privacy and confidentiality reasons, they are not readily available; requests to address the datasets should be directed to oliver.klaproth@airbus.com.

## Ethics Statement

The studies involving human participants were reviewed and approved by the Ethik-Kommission Fakultät V – Verkehrs – und Maschinensysteme Institut für Psychologie und Arbeitswissenschaft TU Berlin. The patients/participants provided their written informed consent to participate in this study.

## Author Contributions

OK, CV, LK, TZ, and NR designed the experiment. CV designed the flight task. LK and TZ designed the EEG trainings. OK and NR created the cognitive model. MH created the interface between flight simulator data and ACT-R. OK, CV, and LK performed the experiments and drafted the manuscript. OK and LK analyzed the data. OK, CV, LK, MH, TZ, and NR edited, revised, and approved the manuscript. All authors contributed to the article and approved the submitted version.

## Conflict of Interest

OK was employed by Airbus Central R&T. CV was employed by Airbus Defence and Space. LK and TZ were employed by Zander Laboratories B.V. The remaining authors declare that the research was conducted in the absence of any commercial or financial relationships that could be construed as a potential conflict of interest.

## Footnotes

1 http://act-r.psy.cmu.edu/

2 The “two streams hypothesis” (Milner and Goodale, 2008) is implemented for the visual and aural module, resulting in a visual- and aural-location buffer for the where-pathway and visual and aural buffers for the what-pathway in the respective modules.

3 http://home.flightgear.org/

4 NAV mode is a managed lateral navigation mode of the autoflight system in which the aircraft follows the flight plan programmed in the FMS.

5 Abbreviated callsign, spoken FOXTROT UNIFORM OSCAR.

6 The spurious electrical alert would vanish by itself irrespective of any flight crew action; the normative response to a TCAS TA alert is to visually acquire the intruding aircraft and to prepare for a subsequent evasive maneuver, should a so-called “Resolution Advisory (RA)” alert follow.
